# Review: Hormone Pregnancy Tests Were Teratogenic by the Same Failed Abortion and Hypoxia‐Related Mechanism as Misoprostol

**DOI:** 10.1002/bdr2.2462

**Published:** 2025-03-14

**Authors:** Bengt R. Danielsson, Helen E. Ritchie

**Affiliations:** ^1^ Division of Clinical Pharmacology Karolinska Institute Stockholm Sweden; ^2^ School of Medical Sciences, Faculty of Medicine and Health University of Sydney Sydney New South Wales Australia

**Keywords:** failed abortion, hormone pregnancy tests, hypoxia, malformation, misoprostol, vascular disruption

## Abstract

**Introduction:**

Hormone pregnancy tests (HPTs), containing synthetic progesterone and oestrogen, were used to diagnose pregnancy in the 1950s to early 1980s. An existing pregnancy was purported to be unaffected while expulsion of the uterine lining (withdrawal bleed) was supposed to occur if the woman was not pregnant. However, studies in the 1960s–1980s associated HPTs with teratogenicity and some countries banned their use in the early 1970s. Following renewed scientific and political interest, studies were published from 2014–2023.

**Materials and Methods:**

This review evaluates whether HPTs fulfil scientific criteria to be teratogenic based on results in old and newer human, animal and mechanistic studies.

**Results and Discussion:**

The evaluation shows that HPT teratogenicity is identical to the established human teratogen misoprostol, with limb reductions, neural tube defects and urinary‐renal system defects as the most significant. The evaluation also presents evidence for abnormal uterine contractions and failed abortion (but the embryo survives) and hypoxia/ROS‐related damage (including vascular disruption) in the embryo secondary to compression of uterine/embryonic vessels, as underlying the teratogenicity. Animal studies show human malformations associated with HPTs could be induced by a single period of embryonic hypoxia, and that HPTs have both abortive and teratogenic potential.

**Conclusion:**

Altogether, HPTs fulfil criteria to be characterised as a human teratogen.

## 1 Introduction

Hormone pregnancy tests (HPTs), such as Primodos in the UK (trade name Duogynon in Germany), were used extensively during the late 1950s to the early 1980s. HPTs contained a high dose of synthetic progesterone, for example, norethisterone (NET) or ethisterone (ET), and a low dose of a synthetic estrogen and were used to diagnose pregnancy. HPTs were given orally for 2 days, resulting in a high peak and then discontinuation of the synthetic progesterone, mimicking the cause of the monthly withdrawal bleed in the non‐pregnant uterus.

The level of uterine activity in humans, in both the non‐pregnant (Pulkkinen 1983) and pregnant uterus (Bygdeman and Swahn 1985; Csapo et al. 1973), is regulated by the balance between the intrinsic stimulator prostaglandin contracting the uterus and the intrinsic suppressor, progesterone, relaxing the uterus. During the menstrual cycle, after ovulation (approximately day 14), the corpus luteum in the ovary releases progesterone. If the egg is not fertilized, the corpus luteum breaks down, the production of progesterone falls, and the decrease in progesterone receptor stimulation causes uterine contractions and expulsion of the endometrial lining. The amplitude of uterine contractions increases dramatically (from < 30 to 50–200 mm Hg) producing labor‐like contractions at the time of menstruation/bleed (Aguilar and Mitchell 2010). If the egg is fertilized, the corpus luteum continues to produce progesterone until the placenta takes over production at around weeks 8–12 of human pregnancy. Progesterone levels continuously increase during pregnancy but may vary considerably between pregnant women. In addition to preventing abnormal uterine contractions, progesterone suppresses prostaglandin production, contributing to keeping the uterus in a quiescent state during organogenesis (Rosen and Yogev 2023).

The instructions from the Primodos manufacturer were that a woman take *“*1 Tablet on each of two consecutive days. Bleeding follows in 3‐6 (rarely as long as ten) days, if there is no pregnancy. An existing pregnancy is unaffected by Primodos.*”* However, several studies published in the late 1960s to 1980s in different European countries and the USA indicated an increased risk of teratogenicity associated with use of HPTs, as briefly presented below and in detail in Sections 3.1, 3.3. Accumulating concerns over teratogenicity led to regulatory warnings and ban of the use of HPTs by 1970 in some countries such as Sweden and Norway. However, although warnings appeared in the UK in 1975, Primodos was not taken off the market until 1978 and Duogynon remained on the market until 1981 in Germany (Heneghan et al. 2018). Following renewed and ongoing scientific and political interest in this topic triggered by alleged victims of HPTs in the UK and Germany seeking recognition, more recent studies on HPTs and teratogenicity have been conducted between 2014 and 2023. A report reviewed epidemiological studies from 1967 and onwards (Seabroke 2017). An increased risk for limb reductions, neural tube defects, orofacial clefts, esophageal atresia/fistula, VACTERL association, urogenital–renal, and cardiovascular defects were reported in the studies (see also Table 1 in Section 3.1.1). By using a quality score system, only limb reductions, esophageal atresia and heart anomalies were assessed robust enough to perhaps represent causal associations, but were considered more likely to have been caused by chance (Seabroke 2017). In contrast, a meta‐analysis reviewing essentially the same epidemiological studies indicated a causal relation and concluded a significant increased risk for VACTERL and malformations in the nervous, musculoskeletal, and gastrointestinal systems (Heneghan et al. 2018).

A German (Tümmler et al. 2014) and a UK study (Beynon and Seabroke 2017) evaluated the rates of malformations in case reports (around 400 and 235, respectively), not previously evaluated versus expected rates based on data in European and British birth registers. An increase in neural tube defects, cleft lip/palate, and defects of extremities (particularly limb reductions) were observed in both studies. The results in the German study suggested an association, but due to possible confounding, no firm conclusions could be drawn. The UK study concluded there was no evidence of an identifiable congenital anomaly or pattern of anomalies associated with HPT exposure. In contrast, a recent mechanistically oriented review (Danielsson et al. 2023) suggested that both HPTs and misoprostol cause a similar pattern of human anomalies as can be produced in animals by transient embryonic hypoxia and generation of reactive oxygen species (ROS).

Misoprostol is a prostaglandin and abortifacient drug inducing uterine contractions. However, when abortion fails and pregnancy continues, misoprostol can induce teratogenicity, as acknowledged worldwide in regulatory pregnancy texts. The teratogenicity has been attributed to uterine contractions, compression of uterine and embryonic vessels (e.g., (Vargas et al. 2000), and periods of embryonic hypoxia and ROS (Chernoff and Rogers 2010; Danielsson et al. 2023).

The purpose of this study is to evaluate how well HPTs fulfill scientifically accepted criteria (based on criteria by Shepard) to be considered teratogenic in humans based on an integrated evaluation of human, animal, and mechanistic studies (Shepard 1994). The same eight criteria were applied when misoprostol was judged to be a human teratogen at an expert‐group meeting with teratologists and epidemiologists (Philip et al. 2003).

## 2 Materials and Methods

### 2.1 Rationale for Approach

The evaluation of human teratogenicity is a rigorous process that involves more than establishing that malformations are substantially and statistically higher in exposed versus nonexposed pregnancies. Long before evidence‐based medicine became a popular term, the pioneers of clinical teratology applied principles of teratology, epidemiology, and causal thinking to establish teratogenicity (Holmes 1999; Shepard 1994). Most established human teratogens have been identified by the clinical recognition of characteristic patterns of congenital anomalies in children of exposed mothers by alert clinicians and not by epidemiological studies (Carey et al. 2009; Källén 2016). Conventional epidemiological studies are designed to permit rigorous statistical assessment, but such investigations usually classify congenital anomalies without adequate consideration of their known etiological heterogeneity. A basic principle in teratology is that both the type and severity of induced malformations can vary with the stage of exposure to human teratogens (Alwan and Chambers 2015). For example, temporary embryonic hypoxia, the proposed teratogenic mechanism behind misoprostol, can cause a wide spectrum of malformations based on the stage of exposure, severity, and duration (Danielsson et al. 2023; Webster and Abela 2007).

The concept of biologic plausibility is central to the discussion of evidence (Carey et al. 2009; Shepard 1994). The understanding of the pathogenesis involved in inducing the effects of the exposure provides biologic plausibility. Understanding the potential mechanisms of drug action and identifying specific patterns of malformations contribute to the assessment that a particular cause‐and‐effect relationship is biologically plausible. As expressed in regulatory EU guidelines on the evaluation of human (Committee for Medicinal Products for Human Use (CHMP) 2019) and animal teratogenicity (ICH S5 (R3) 2020), there are many examples where the pharmacological effect of the medicine is related to the mechanism of teratogenicity. Therefore, when assessing teratogenicity signals for a drug, it is crucial to compare, in detail, adverse pregnancy outcomes, including malformation patterns, with those of a known teratogen having similar pharmacological effects.

The EUROCAT (European Platform on Rare Disease Registration) birth register characterizes subgroups of major malformations that share common etiologic or clinical characteristics. The system considers the balance needed between “lumping” together heterogeneous sets of anomalies and “splitting” so finely that there are few cases in each group. Lumping can lead to inaccurate estimates and missed detection of specific malformations within an organ system.

These registers formed groups of anomalies that are realistically available in medical records and regional or national databases, and which can be diagnosed with high precision and accuracy for coding. In EUROCAT today, 100 subgroups of major malformations are characterized (Bergman et al. 2024). It is possible to study an increase in subgroups of malformations that share common etiologic characteristics in an organ system without lumping together heterogeneous anomalies.

Specific criteria and guidelines for evaluation of human teratogenicity, which take in consideration the above aspects have been established and accepted in the scientific community. The criteria used to evaluate HPTs in this study are the same criteria as used when misoprostol was judged to be a human teratogen by a group of experts in teratology and epidemiology (Philip et al. 2003). The criteria are based on Shepard's criteria for “proof” of human teratogenicity (Shepard 1994) with some modifications (Philip et al. 2003). The criteria listed below are not diagnostic algorithms but rather a set of relevant considerations for making a scientific judgment on whether a certain exposure is likely to cause human malformations. It is important to note that most known human teratogens do not present positive evidence for every factor, for example, misoprostol did not fulfill criteria 7 below. The same type of judgment based on integration of available clinical, animal data and mechanistic information is used by regulatory agencies in pregnancy labeling, for example, “Guideline on risk assessment of medicinal products on human reproduction and lactation: from data to labelling” in the EU (Committee for Medicinal Products for Human Use (CHMP) 2009).

Three criteria are considered to be “essential,” that is, must be fulfilled (marked with *). The other criteria are characterized as “helpful” or “supportive” to strengthen an association as causal:

1. Specific defect(s) could be identified by careful delineation of clinical case reports.*
2. Consistency in results between epidemiological studies.*
3. Substantially and statistically higher prevalence of anomalies in exposed versus non‐exposed pregnancies.
4. Recognizable pattern of malformations.
5. Exposure to agent at critical time(s) in development.*
6. Biological plausibility and mechanistic information.
7. Increased incidence of anomalies in a population after the introduction of the agent.
8. Animal studies–human developmental effects can be recreated in animal models.

## 3 Results and Discussion

### 3.1 Clinical Evaluation of the Observed Manifestations of HPT‐Related Teratogenicity by Applying Criteria 1–3

The first three criteria will be discussed together as they all relate to clinical studies. In the current study, criterion 1, “specific defect(s),” is defined as a malformation, which in the EUROCAT system is classified as a congenital anomaly subgroup. A “substantial increase” (criterion 2) is defined as a 2‐fold increase, or alternatively a statistically significant increase of a “specific defect.” Criterion 3 “consistency in results” is defined as at least two epidemiological studies by two different research groups and/or in two different populations showing a “substantial increase” in “specific defects.”

The purpose of this section is to present data on human malformations associated with HPTs and evaluate if any type of malformation(s) fulfill criteria to suggest a causal association according to the criteria system. The analyses encompass and provide commentary on data from “older studies” published largely in the 1970s and 1980s (Section 3.1.1) and in a newer study, including a large study based on not previously evaluated case reports (see Section 3.1.2). Finally, Section 3.1.3 presents an integrated assessment of malformations from both the “older” and newer studies to evaluate if any “specific defect(s)” fulfill all three clinical criteria, thereby indicating causality.

#### 3.1.1 Results and Comments on “Older” Epidemiological Studies

The “older” studies consisted of more than 25 studies (case–control or cohort studies) presented in Table 1; this table is completely based on a public report by an epidemiological assessor at the UK regulatory agency (Seabroke 2017). The report reviewed the results in human HPT studies from 1968 to 2014 by using a quality scoring system. The studies selected for quality evaluation were based on a publicly available comprehensive report based on a literature search (using specific key words etc.) by another regulatory UK assessor (Datta‐Nemdharry 2016) and also applied inclusion criteria by Seabroke in 2017. For example, studies indicating an increase in specific malformations, for example, limb reduction defects (Jaffe et al. 1975; Papp et al. 1976; Robertson‐Rintoul 1974) without any control/comparator were not included.

**TABLE 1:** HPT‐associated malformations showing a > 25% increase in at least one study (Seabroke 2017).

Major limb reductions	
(Lammer and Cordero 1986) USA^b^	2.16*
(Vajda and Czeizel 1976) Hungary	1.32
(Oakley, Flynt, and Falek 1973) USA	1.94
(Janerich, Piper, and Glebatis 1974) USA	3.06
(Hellström et al. 1976) Sweden	2.90
Orofacial clefts	
Cleft lip ± cleft palate	
(Oakley, Flynt, and Falek 1973) USA	1.4
(Lammer and Cordero 1986) USA	0.95
(Tümmler et al. 2014)	1.59*
Cleft palate	
(Oakley, Flynt and Falek 1973) USA	1.67
(Lammer and Cordero 1986) USA	1.28
Cardiovascular anomalies (all types)	
(Janerich et al. 1977) USA	5.43*
(Heinonen et al. 1977) USA	2.1*
(Nora et al. 1978) USA^a^	3.55–6.0*
(Hadjigeorgiou et al. 1980) Greece	3.34
(Torfs, Mikovich, and Van Den Berg 1981) USA	0.61
(Rothman et al. 1979) USA	1.3
(Goujard and Rumeau‐Rouquette 1977) France	1.05
(Goujard, Rumeau‐Rouquette, and Saurel‐Cubizolles 1979) France	1.49
(Boldt 1977) Germany	1.79
Transposition of great vessels	
(Levy, Cohen and Fraser 1973) USA	3.04
Digestive and abdominal wall defects	
Oesphageal atresia/fistula	
(Lammer and Cordero 1986) USA	2.81*
(Oakley, Flynt, and Falek 1973) USA	4.13
Intestinal atresia	
(Oakley, Flynt, and Falek 1973) USA	1.29
Omphalocele	
(Oakley, Flynt, and Falek 1973) USA	1.47
Diaphragmatic hernia	
(Lammer and Cordero 1986), USA	1.47
(Oakley, Flynt, and Falek 1973) USA	1.22
Nervous system anomalies (all types)	
(Torfs, Mikovich, and Van Den Berg 1981) USA	3.80*
(Roussel 1968) UK	1.27
Neural tube defects (all types)	
(Tümmler et al. 2014) Germany	2.99*
(Oakley, Flynt, and Falek 1973) USA	0.97
Spina bifida	
(Gal 1972) UK	5.63*
(Lammer and Cordero 1986) USA	1.02
(Oakley, Flynt, and Falek 1973) USA	1.26
Spina bifida and anencephaly	
(Sainz, Rodríguez Pinilla, and Martínez Frías 1987) Spain	8.57
(Laurence et al. 1971) UK	1.26
(Gaal and Ukvari 1977) UK	0.83
Encephalocele	
(Lammer and Cordero 1986) USA	1.49
Microcephaly	
(Goujard and Rumeau‐Rouquette 1977) France	5.62*
(Goujard, Rumeau‐Rouquette, and Saurel‐Cubizolles 1979) France	25.6*
Renal and urinary defects	
Unilateral absence kidney	
(Tümmler et al. 2014) Germany	2.53*
Bilateral renal hypoplasia	
(Goujard, Rumeau‐Rouquette, and Saurel‐Cubizolles 1979) France	25.6*
Bladder exstrophy	
(Tümmler et al. 2014) Germany	37.3*
Urogenital defects (all types)	
(Torfs, Mikovich, and Van Den Berg 1981) USA	2.03
Hypospadias	
(Mau 1981) Germany	2.19
Skeletal defects	
(Tümmler et al. 2014) Germany	1.95*
(Goujard and Rumeau‐Rouquette 1977) France	0.95
VACTERL association	
(Nora and Nora 1975) USA^a^	9.74–13.5*
(Nora et al. 1978) USA	8.41*
(Lammer, Cordero and Khoury 1986), USA^a^	0.93–0.98

*Note:* Effect Estimate increased (> 1.0) or decreased (< 1.0) of malformations in HPT exposed pregnancies compared to controls.

* Statistically significant difference.

^a^ More than one analysis in the same study.

^b^ Research group lead author.

The quality evaluation by Seabroke (2017) only assessed methodological aspects, for example, confounding, selection of controls, exposure ascertainment, sample size, and multiplicity, etc., in relation to modern standards of epidemiology, but not aspects investigated in the current study, for example, recognizable patterns of malformation or biological plausibility based on mechanistic information. Summary statistics for each study were presented using forest plots, and when not available from the original study, these were calculated from proportion data (Seabroke 2017). As can be seen in Table 1, the subtypes of anomalies showing at least a 2‐fold increase included limb reduction defects, neural tube defects, microcephaly, esophageal atresia/fistula, transposition of great vessels, unilateral absence of kidney, and urine bladder exstrophy. In addition, VACTERL association was increased more than 5‐fold. VACTERL is a rare multiple congenital anomaly (prevalence is 1 per 10,000–40,000 live births) characterized by the presence of at least three of the following malformations: vertebral defects, anal atresia, cardiovascular defects, tracheoesophageal fistula and/or esophageal atresia, renal anomalies, and limb abnormalities, such as radial‐type limb reductions (Carli et al. 2014; Czeizel and Ludányi 1984; La Placa et al. 2013; Solomon 2011).

In the current study, by applying the criteria system, four specific subgroups of anomalies suggest a causal association with HPTs: limb reduction defects, neural tube defects, esophageal atresia and cleft lip ±cleft palate. In addition, cardiovascular defects, on the organ system level, also fulfill criteria 1–3. Three of the anomalies identified by using this criteria system (limb reductions, esophageal atresia and cardiovascular anomalies), also stood out in the quality score assessment of the “older” HPT studies in Table 1 (Seabroke 2017). The author concluded that the “older” studies listed in Table 1 “generally have limitations in relation to study design due to being conducted many decades ago when epidemiological methods were not as advanced as they are today” (Seabroke 2017). However, although limb reductions, esophageal atresia, and heart data were considered to be robust enough to represent a causal association, the author concluded that the association was more likely to have been caused by chance.

#### 3.1.2 Results in Newer Epidemiological Studies Conducted After 2016

A meta‐analysis essentially reviewing the same “old” studies as in Table 1 (in total 16 case–control and 10 prospective cohort studies) found a statistically significant 2‐fold or higher increased risk for nervous, musculoskeletal, gastrointestinal, and cardiovascular malformations on the organ system level as well as the VACTERL association (Heneghan et al. 2018). In contrast to the earlier report, these authors concluded that there was a causal association between HPTs and human teratogenicity (Heneghan et al. 2018).

A further study by two epidemiological assessors at the UK regulatory agency investigated the evidence of a causal association of malformations and HPTs in a cohort of more than 200 case reports not previously evaluated (Beynon and Seabroke 2017). The reported anomalies in the case reports are presented in Table 2. The case reports originated mainly from regulatory bodies (UK, Australia, New Zealand and WHO) and a patient organization in the UK. These exposures had occurred before HPTs were withdrawn from the market. Subgroups of major malformations in the case reports were coded according to criteria in the EUROCAT birth register before analysis. This report is the most up‐to‐date study existing in relation to outlining all specific defects/subgroups associated with HPT use in pregnancy. The report compared changes in the proportions of malformations in the HPT cohort in relation to the expected rate based on data in European birth registers (EUROCAT). This type of methodology is today used as signal detection to identify unusual associations between medications and congenital anomalies (Dolk 2015).

**TABLE 2:** Compilation of proportional reporting ratios for observed single major malformations in the HPT‐exposed dataset compared with the EUROCAT register.

Organ system(s) affected (bold)	HPT case^b^ (*n*)	HPT use proportion of all anomalies (%)^b^	Anomalies HPT versus EUROCAT × fold difference^b^	Calculated proportion of all anomalies in the general population (%)^c^	Subtypes usually identified at birth or first week after birth^d^	Calculated prevalence of anomaly per 10,000 births (%)^e^
Subtypes of malformations following EUROCAT criteria						
**Limbs**	52	31.7	1.8	17.6		42.1 (0.42)
Limb reduction defect^a^	28	17.1	7.4*	2.3	Yes	5.5 (0.06)
Club foot	6	3.7	0.8	4.6	Yes	11.1 (0.11)
Syndactyly	5	3.0	1.7	1.8	No	4.6 (0.04)
**Nervous system** ^a^	37	22.6	2.1*	10.3		24.6 (0.25)
Neural tube defect^a^	16	9.8	2.1*	4.7	Yes	11.2 (0.11)
Encephalocele^a^	3	1.8	3.6*	0.5	Yes	1.2 (0.01)
Anencephaly	1	0.6	0.3	2.0	Yes	4.8 (0.05)
Spina bifida^a^	12	7.3	3.2*	2.3	Yes	5.5 (0.05)
Hydrocephalus	4	2.4	1.0	2.4	Yes	5.7 (0.06)
Microcephaly	2	1.2	1.2	1.0	No	2.4 (0.02)
**Eyes** ^a^	9	5.5	3.4*	1.6		3.9 (0.04)
Microphthalmos^a^	3	1.8	6*	0.3	No	0.7 (0.01)
Anophthalmus^a^	3	1.8	18*	0.1	Yes	0.2 (0.002)
Congenital glaucoma^a^	1	0.6	6	0.1	No	0.2 (0.002)
Congenital cataract	1	0.6	1.5	0.4	No	1.0 (0.004)
**Orofacial region**	18	11.0	1.8	6.1		14.6 (0.15)
Cleft lip ± cleft palate^a^	14	8.5	2.2*	3.9	Yes	9.2 (0.09)
Cleft palate	4	2.4	1.0	2.4	Yes	5.7 (0.06)
**Cardiovascular system**	38	23.2	1.0	23.2		55.4 (0.55)
Severe cardiac disease	8	4.9	0.5	9.8	No	23.4 (0.23)
Transposition great vessels^a^	3	1.8	2.3	0.8	Yes	1.9 (0.02)
Ventricular septum defects	5	3.0	0.3	10.0	No	23.9 (0.24)
Atrial septum defects	1	0.6	0.2	3.0	No	7.2 (0.07)
Tetralogy of Fallot	2	1.2	1.1	1.1	No	2.6 (0.03)
Tricuspid atresia/stenosis^a^	2	1.2	4.8	0.3	Yes	0.6 (0.01)
Pulmonary valve stenosis	1	0.6	0.5	1.2	No	2.9 (0.03)
Hypoplastic right heart^a^	1	0.6	2*	0.3	Yes	0.7 (0.01)
Coraction aorta	1	0.6	0.4	1.5	No	3.6 (0.04)
**Digestive system**	15	9.1	1.2	7.6		18.1 (0.18)
Esophageal atresia/fistula	3	1.8	1.8	1.0	Yes	2.4 (0.02)
Ano‐rectal atresia/stenosis^a^	6	3.7	3.1*	1.2	Yes	2.9 (0.03)
**Urinary system**	10	6.1	0.6	10.2		24.3 (0.24)
Bladder exstrophy^a^	1	0.6	2	0.3	Yes	0.7 (0.01)
**Genital region**	8	4.9	0.7	7.0		16.7 (0.17)
Hypospadias	4	2.4	0.5	4.8	No	11.5 (0.11)
**Lateral anomalies** ^a^	4	2.4	2.7	0.9	No	2.1 (0.02)
**Situs inversus** ^a^	2	1.2	5*	0.2	No	0.6 (0.01)
**(Vascular disruption)** ^a^	13	7.9	2.3*	3.4	Yes	8.2 (0.08)

* Statistical significance is based on 95% confidence intervals presented in Beynon and Seabroke (2017).

^a^ Increases in proportions two times or more.

^b^ Table H (Beynon and Seabroke 2017).

^c^ % HPT use as a proportion of all anomalies/%EUROCAT proportion of all anomalies in the general population.

^d^ (EUROCAT 2013).

^e^ Calculated prevalence data. 2.39 (prevalence of major anomalies per 10 000 birth) x % EUROCAT proportion of all anomalies in general population.

EUROCAT also notes whether the malformation can be detected at birth or usually not detected until more than 1 week after birth (EUROCAT 2013). This is important as anomalies not obviously detected at birth by visual inspection or due to severe symptoms are likely to be detected to a higher extent today due to better techniques (e.g., ultrasound) and better surveillance of pregnancies compared to the late 1950s to late 1970s when HPTs were used. For example, the prevalence of diagnosed live‐born with cardiac defects in Sweden has tripled during 1970–2017 (Giang et al. 2023). Whereas the most complex cardiovascular lesions have increased slightly, remained stable, or declined, mild lesions such as ventricular and atrial septal defects have increased dramatically over time most likely reflecting improved diagnosis (Giang et al. 2023). Similarly, the prevalence of hypospadias has increased dramatically from around 1970 to the 2000s (Dolk 2004; Leunbach et al. 2025).

In contrast, it is highly likely that malformations, which are obvious to detect at birth according to EUROCAT, would be detected to a similar extent in pregnancies in the late 1950s to late1970s as in more recent birth registers covering the years 1980–2014. It is therefore essential to take this aspect into consideration. This might explain why four less severe (not life threatening) cardiovascular defects, including atrial and ventricular defects, and hypospadias were reported to be decreased in HPT‐exposed pregnancies in the case report series (Beynon and Seabroke 2017). Whether a malformation can usually be detected at birth as well as data on the prevalence of anomalies in the general population has therefore been included in Table 2. This information was not included in the case report series (Beynon and Seabroke 2017); otherwise, all data in Table 2 are based on this report.

The subgroups of major anomalies showing a > 2‐fold proportional increase in HPT pregnancies (in total 18) compared to the EUROCAT registers are compiled in Table 2. Of these, 12 anomalies were both detectable at birth and > 2‐fold “substantially increased”; 10 out of the 12 malformations detectable at birth were also statistically significantly increased based on confidence intervals (Beynon and Seabroke 2017). The two most common malformations observed in HPT‐exposed pregnancies, limb reductions (7.4‐fold increase) and spina bifida (3.2‐fold increase), were statistically significantly increased. In addition to malformations showing a > 2‐fold increase, about 30 different single or combined defects were observed.

The case report series (Beynon and Seabroke 2017) also listed malformations in children with multiple anomalies associated with the use of HPTs. The defects were listed in free text, and several were not possible to code according to EUROCAT (e.g., “malformation of heart, unspecific”). The proportion of children with multiple major defects was higher than expected (19%) compared to EUROCAT data (8%) but no commentary was provided (Beynon and Seabroke 2017). However, combined defects are of high interest since multiple defects in an individual are likely to have been caused by the same teratogenic mechanism (Friedman 1992). Analysis of the cases with multiple defects in the current study showed 7/44 (16%) had combined nervous system and limb defects, seven (16%) cranial/oromandibular defects, and 13 (30%) had cardiovascular defects combined with other major anomalies. Also, seven (16%) showed unilateral renal agenesis combined with other defects. Unilateral renal agenesis was updated to be a major malformation in EUROCAT (2023) but was not classified as such at the time of the report by Beynon and Seabroke (2017). Furthermore, 8 (18%) of the children had multiple malformations consistent with VACTERL association. The birth prevalence of unilateral renal agenesis is 4.0/10,000 (Laurichesse Delmas et al. 2017) and VACTERL association is 1 per 10,000 to 40,000 live births, as mentioned previously.

These findings indicate that both unilateral renal agenesis, as well as the combined anomalies mentioned above, were substantially increased in the HPT data set in the study. In addition, among the case reports, there was also one case each of Moebius syndrome and Klippel–Feil syndrome. These syndromes are also very rare (prevalence around 1 per 10–40,000 live births) but are important as the most prevalent theory of their etiology has been related to embryonic hypoxia and vascular disruption (Bavinck and Weaver 1986).

Vascular disruption defects were also increased. However, vascular disruption is a not a term used prior to the early 1980s and is rather a mechanism for a spectrum of hypoxia‐related malformations, including limb reductions and several nervous system defects in humans (Van Allen 1981, 1992). It is therefore not possible to compare vascular disruption defect rates between the “older studies” conducted mainly in the 1970s and 1980s (Table 1) and a newer study (Table 2).

#### 3.1.3 Evaluation of HPT Teratogenicity by Integrating Results in Both “Old” and Newer Studies

The subtypes of anomalies reported to be substantially increased in both the “old” (Section 3.1.1) and more recent studies (Section 3.1.2) substantially overlap. If data from both the older studies and the new studies are evaluated altogether, the results show that several “carefully delineated specific defects” were “substantially increased” (at least 2‐fold increased or showed a statistically significant increase) in a “consistent” manner (in at least two epidemiological studies by two different research groups and/or in two different populations) thereby fulfilling criteria to be causally related with HPTs. The anomalies where available data suggest a causal relation with HPT use based on criteria 1–3 are discussed below.

*Limb reduction defects*: All five “old studies” in Table 1 (Section 3.1) showed an increase in limb reduction defects. Four out of the five studies showed a 2‐ to 3‐fold increase; one of those also showed a statistically significant increase. The quality of the data in these “old” studies was considered robust enough to ascribe a causal association with increased limb reductions (Seabroke 2017). The newer case report study showed a 7.4‐fold increase in limb reductions in HPT‐exposed pregnancies versus the EUROCAT birth register (see Table 2) and a 13‐fold increase versus the BINOCAR (British and Northern Ireland) birth register (Beynon and Seabroke 2017). The BINOCAR data are not presented in Table 2 but were also analyzed in the same way as EUROCAT data in this report (Beynon and Seabroke 2017).

*Esophageal atresia with or without tracheoesophageal fistula*. Both the two “old” studies evaluating this type of malformation showed a more than 2‐fold increase; one showed a statistically significant increase of around 3‐fold and the other a 4‐fold increase (see Table 1). The quality of the data in these “old” studies was considered robust enough to ascribe a causal association with increased esophageal atresia/fistula (Seabroke 2017). The large case series study reported a 3‐fold increase versus the BINOCAR birth register (Beynon and Seabroke 2017).

*Neural tube defects, including the “sub‐subgroups” encephalocele and spina bifida*. One of the two “old” studies evaluating neural tube defects (all types) showed a more than 3‐fold significant increased risk (Table 1). In relation to subtypes of neural tube defects in the “old” studies, 5 of 7 studies report a more than 25% increase in HPT‐exposed pregnancies; two studies reported a > 2‐fold increase, and one study showed a significant increased risk (Table 1). In addition, the large case report study reported a statistically significant increased risk for neural tubes (2‐fold), spina bifida (3‐fold) and encephalocele (3.6‐fold) versus EUROCAT in HPT‐exposed pregnancies (Beynon and Seabroke 2017; Table 2). Spina bifida (2.7‐fold) and encephalocele (3‐fold) were also increased compared to the BINOCAR register.

*Cleft lip with or without cleft palate* was increased in two out of three old studies (Table 1), with one statistically significant increase (1.6‐fold). This anomaly was also statistically significantly increased 2.2‐fold versus EUROCAT the case report study and 2.5‐fold increased versus the BINOCAR birth register (Beynon and Seabroke 2017; Table 2).

*Transposition of great vessels* showed a 3‐fold increase in the only “old” study focusing on this anomaly (Table 1). This anomaly was also statistically significantly increased 2.3‐fold in comparison with EUROCAT (Table 2) and increased 11‐fold compared with the BINOCAR birth register, the case report study (Beynon and Seabroke 2017).

*Urinary bladder exstrophy* was statistically significantly increased (37‐fold) in the only “old” studies investigating this anomaly (Tümmler et al. 2014) Germany) and also showed an approximately 3‐fold increase in HPT‐exposed pregnancies versus both the EUROCAT and BINOCAR registers (Beynon and Seabroke 2017).

*VACTERL association* was reported to be more than a 5‐fold increase in two “old” studies by the same research group (Table 1). A meta‐analysis based on 26 “old” studies (essentially the same as in Table 1) showed a statistically significant increase in VACTERL (Heneghan et al. 2018). In addition, when we reviewed the multiple malformations in the case reports (Beynon and Seabroke 2017), a substantial increase in VACTERL association was recognized (see Section 3.1.2 for details). However, no syndromes/associations based on cases with multiple malformations were investigated in the case report study (Beynon and Seabroke 2017).

*Unilateral kidney agenesis* was reported to be significantly increased 2.5‐fold in the only “old” studies investigating this anomaly (Tümmler et al. 2014, Germany). However, as with VACTERL, when we reviewed the multiple malformations listed in the case report study (Beynon and Seabroke 2017), a substantial increase in unilateral kidney agenesis was identified. Unilateral kidney agenesis was not investigated in the case report study (Beynon and Seabroke 2017) (see Section 3.1.2 for details).

#### 3.1.4 Concluding Remarks Related to Teratogenicity by Applying Criteria 1–3

Altogether, the integrated evaluation applying clinical criteria on conducted studies from different countries by different researchers over an extended period indicates a causal association between HPT exposure and an increased risk for HPT teratogenicity related to the above subtypes of malformations. This is a different conclusion than that reached in the reports from the “old” (Seabroke 2017) and newer (Beynon and Seabroke 2017) studies and in a summary report assessing evidence for human teratogenicity by HPTs based on these reports (Commission on Human Medicines 2017). By analyzing the data in each report separately, the essential consistency between the studies was missed.

The report of “old” studies (Table 1; Seabroke 2017) only assessed methodological quality. Even malformations (e.g., limb reductions) where the “old studies” were considered to be of robust quality enough to indicate a causal association were interpreted to be more likely to have been caused by chance. The case series report (Beynon and Seabroke 2017) focused on the characterization of malformations in HPT pregnancies, showing an increase versus the EUROCAT and BINOCAR registers, and dismissed the consistency in results between the “older” studies and their case report study. The authors concluded that there was no evidence of an identifiable congenital anomaly or pattern of anomalies associated with HPTs. However, as will be shown in Section 3.2, the observed pattern of HPT malformations almost completely agrees with the reported pattern for the established human teratogen misoprostol when abortion fails.

### 3.2 Criterion 4: Evaluation of a Recognizable Pattern of Malformations in Clinical Studies

Guidelines in the evaluation of teratogenicity stress the importance of comparing the pattern of malformations for a suspected teratogen (in this case HPTs) with an established human teratogen (misoprostol), if the suspected teratogen may exert the same pharmacological effect (in this case uterine contractions in organogenesis) as the established teratogen (Committee for Medicinal Products for Human Use (CHMP) 2019). As mentioned in the Introduction, a brief comparison was performed in a recent mechanistic‐oriented study; the results suggested a similar spectrum of malformations between HPTs and misoprostol (Danielsson et al. 2023). The current study provides additional and more extensive information on teratogenicity in studies with misoprostol, including detailed information on observed increases of specific malformations in individual studies using the same EUROCAT terminology for HPTs as for misoprostol in characterizing the defects.

In total, 21 publications (5 case control studies, 3 cohort studies, 10 case report series and 3 single case reports on single or more common combined malformations in a child) after misoprostol use in the first trimester were reviewed. In total, reported malformations in 253 cases were evaluated (Table 3). The great majority of cases were reported in connection with failed abortion after the use of misoprostol to induce abortion, but a few were observed after the use of misoprostol to prevent peptic ulcer. The studies were reported from different countries, including Brazil, Argentina, the Philippines, France, and the US. An overall 2–4 times increased risk for teratogenicity after misoprostol use was reported in several studies (Barbero et al. 2011; Brasil et al. 2000; Dal Pizzol et al. 2006, 2008; Opaleye et al. 2010). Two large studies were focused on two specific subgroups of anomalies: limb reductions and cranial nerve palsy (Moebius syndrome). Both reported an increased risk for limb reduction defects (12‐ and 24‐fold) and Moebius syndrome (25‐ and 49‐fold), respectively (Dal Pizzol et al. 2006; Vargas et al. 2000). A third study only focused on Moebius syndrome; this study showed a 30‐fold increase (Pastuszak et al. 1998).

**TABLE 3:** Misoprostol‐associated major anomalies. Alterations (ORs) compared to controls/birth registers are given in parentheses when such information is available.

Organ systems affected
Subgroups of anomalies using EUROCAT criteria (bold italic)
Limbs
***Limb reduction defects***: Orioli and Castilla (2000) (OR = 12*), Dal Pizzol et al. (2006) (OR = 12*), Vargas et al. (2000) (OR = 25*), (Auffret et al. 2016; Chiong and Cutiongco‐de la Paz 2009; Collins and Mahoney 1983; Genest et al. 1999; Gonzalez et al. 1998; Gonzalez et al. 1993; Opaleye et al. 2010; Orioli and Castilla 2000; Wood et al. 1987)
***Club foot/Equinovarus***: Orioli and Castilla (2000) (OR = 1), (Auffret et al. 2016; Chiong and Cutiongco‐de la Paz 2009; Collins and Mahoney 1983; Dal Pizzol et al. 2008; Gonzalez et al. 1998; Gonzalez et al. 1993; Opaleye et al. 2010; Orioli and Castilla 2000; Vauzelle et al. 2013; Vendramini‐Pittoli et al. 2013)
***Arthrogryposis***: Orioli and Castilla (2000) (OR = 8) (Gonzalez et al. 1998; Opaleye et al. 2010; Orioli and Castilla 2000; Vargas et al. 2000)
***Syndactyly***: Orioli and Castilla (2000) (OR = 2), (Auffret et al. 2016; Chiong and Cutiongco‐de la Paz 2009; Dal Pizzol et al. 2008; Gonzalez et al. 1998; Gonzalez et al. 1993; Opaleye et al. 2010)
Nervous system
***Neural tube defects***: Orioli and Castilla (2000); (OR = 18* and OR = 5 for two types) (Auffret et al. 2016; Barbero et al. 2011; Brasil et al. 2000; Dal Pizzol et al. 2008; Orioli and Castilla 2000; Vauzelle et al. 2013; Vendramini‐Pittoli et al. 2013)
***Hydrocephalus***: Orioli and Castilla (2000) (OR = 4), (Auffret et al. 2016; Chiong and Cutiongco‐de la Paz 2009; Collins and Mahoney 1983; Genest et al. 1999; Gonzalez et al. 1998; Gonzalez et al. 1993; Opaleye et al. 2010; Orioli and Castilla 2000; Wood et al. 1987)
***Microcephaly***: Auffret et al. (2016; Chiong and Cutiongco‐de la Paz 2009; Dal Pizzol et al. 2008; Gonzalez et al. 1998; Gonzalez et al. 1993; Opaleye et al. 2010; Vendramini‐Pittoli et al. 2013)
Eyes
***Ocular anomalies/micro‐anophthalmus***: Chiong and Cutiongco‐de la Paz 2009; Vendramini‐Pittoli et al. 2013
Orofacial clefts
***Cleft lip ± cleft palate***: (Orioli and Castilla 2000; Vauzelle et al. 2013; Vendramini‐Pittoli et al. 2013)
***Cleft palate***: Orioli and Castilla (2000) (OR = 6); (Chiong and Cutiongco‐de la Paz 2009; Gonzalez et al. 1998; Gonzalez et al. 1993; Vauzelle et al. 2013; Vendramini‐Pittoli et al. 2013)
Cardiovascular system
***Life threatening anomalies detected at birth***: Transposition great vessels (Auffret et al. 2016; Ferreira et al. 1999)
***Less severe anomalies usually detected* > *1 week after birth***: (Bernardi et al. 2010; Brasil et al. 2000; Ferreira et al. 1999; Orioli and Castilla 2000; Vauzelle et al. 2013; Vendramini‐Pittoli et al. 2013)
Digestive system
***Esophageal atresia/fistula***: (Bernardi et al. 2010)
***Ano‐rectal atresia/stenosis***: (Bernardi et al. 2010; Opaleye et al. 2010)
***Omphalocele***: (Genest et al. 1999; Opaleye et al. 2010)
Renal and urogenital system
***Bladder exstrophy***: Orioli and Castilla (2000) (OR = 47*)
***Unilateral renal agenesis***: (Auffret et al. 2016; Bernardi et al. 2010; Cheyne et al. 2014)
***Hypospadias***: Orioli and Castilla (2000) (OR = 1.6), Gonzalez et al. 1998; Gonzalez et al. 1993
Combined anomalies and laterality
***VACTRL association***: (Bernardi et al. 2010; Cheyne et al. 2014)
***Combined nervous and limb anomalies***: (Barbero et al. 2011; Chiong and Cutiongco‐de la Paz 2009; Collins and Mahoney 1983; Gonzalez et al. 1998; Gonzalez et al. 1993; Opaleye et al. 2010; Vendramini‐Pittoli et al. 2013)
***Combined cranial/oromandibular defects with other major anomalies*** (Chiong and Cutiongco‐de la Paz 2009; Fonseca et al. 1991; Orioli and Castilla 2000; Vargas et al. 2000; Vauzelle et al. 2013)
***Moebius***: Dal Pizzol et al. (2006) (OR = 12*); Pastuszak et al. (1998) (OR = 12*); Vargas et al. (2000) (OR = 12*); Auffret et al. (2016); Brasil et al. 2000; Cheyne et al. (2014); Chiong and Cutiongco‐de la Paz (2009); Vendramini‐Pittoli et al. (2013)
***Dextrocardia***: Gonzalez et al. (1993)

* Statistically significant increase.

A case–control study (Orioli and Castilla 2000) compiled the rates of birth defects from seven studies up to the year 2000 with misoprostol alone (Collins and Mahoney 1983; Fonseca et al. 1991; Genest et al. 1999; Gonzalez et al. 1998, 1993; Orioli and Castilla 2000; Wood et al. 1987). In total, 29 different anomalies were reported. The odds ratios (OR) of birth defects associated with misoprostol were compared to expected rates in a Latin American birth register in a similar manner to HPTs in Table 2. The results showed that anomalies in the subgroup neural tube defects (OR 5.38–18.06), limb reduction defects (OR 11.68–12.04) and urinary bladder exstrophy (OR 46.83) were significantly increased. Other specific defects that increased included hydrocephalus (4‐fold), orofacial clefts (6‐fold) and syndactyly (2‐fold). The results in the case report series (> 100 children) further characterized the pattern of anomalies associated with misoprostol (without estimating risks for certain anomalies) and confirmed the existence of a pattern of “typical” malformations associated with misoprostol.

As shown in Table 3, the misoprostol‐associated occurrence of subgroups of major anomalies affecting limbs, central nervous system, gastrointestinal system, orofacial clefts, and specific defects affecting the urogenital/renal system is very similar to that reported following HPTs. Table 3 also shows that rare combinations of malformations associated with HPTs, such as VACTERL, Mobius association, combined nervous and limb anomalies, and combined cranial/oromandibular defects with other major anomalies, have also been reported to be associated with misoprostol. In this respect, it is of special interest to note that all the malformations that fulfill the criteria to be causally related with HPTs (limb reduction and neural tube defects, cleft lip/cleft palate, bladder exstrophy, renal agenesis, VACTERL, esophageal atresia/fistula and transposition of great vessels) were also associated with misoprostol use and failed abortion. Furthermore, the most prominent anomalies associated with HPTs (limb reduction defects, neural tube defects and urinary bladder exstrophy), were similarly highly and statistically significantly increased in misoprostol‐exposed pregnancies (Table 4). Altogether, these results show that criterion 4 “recognizable pattern malformations” is fulfilled for HPTs and strongly indicates a common etiology for teratogenicity of HPTs and misoprostol.

**TABLE 4:** Similarities in major malformations fulfilling criteria to be causally related to HPTs (Section 3.1) with those reported for misoprostol (Section 3.2).

Hormone pregnancy tests	Misoprostol
Limb defects	
***Limb reductions*** *	***Limb reductions*** *
Nervous system malformations	
***Neural tube defects*** *	***Neural tube defects*** *
Combined anomalies	
***VACTERL*** *	VACTERL (NC)
Orofacial clefts	
***Cleft lip ± cleft palate*** *	***Cleft lip ± cleft palate***
Gastrointestinal and abdominal wall defects	
***Esophageal atresia/fistula*** *	Esophageal atresia/fistula (NC)
Renal and urogenital system defects	
***Urinary bladder exstrophy*** * ***Renal agenesis*** *	***Urinary bladder exstrophy*** * Renal agenesis (NC)
Cardiovascular defects	
*Life threatening defects detected at birth*: ***Transposition great vessels***	*Life threatening defects detected at birth* Transposition great vessels (NC)

*Note:* Bold italic indicates that anomalies increased by 2‐fold, NC, no data available in relation to alteration compared to controls/birth register data.

* Statistically significant increase in at least one study.

Furthermore, a very brief comparison of the pattern of malformations associated with HPTs and misoprostol seems to also agree with the pattern of malformations of the RCEM disorders (recurrent constellations of embryonic malformations) in which a genetic etiology has not been found (Adam et al. 2020). Adam et al. 2020 suggest that the RCEM group of a conditions should be considered a spectrum with a shared pathogenesis across all phenotypes, with future studies designed to also include maternal factors and markers for prenatal hypoxia.

### 3.3 Criterion 5: Evaluation of Exposure to HPTs at Critical Times in Development

Almost all established human teratogens require that exposure occurs during the period of organ formation (organogenesis). Data on the exact timing of exposure to HPTs were not given in several of the cases in the reviewed studies in Section 3.1. For example, in the case report study (Beynon and Seabroke 2017), exposure data from approximately 70 case reports reported multiple defects; the timing of exposure was given in less than 50% of the cases. All exposures with timing data reported in the study were in the interval 4–12 weeks of pregnancy, with 60% occurring in weeks 6–8.

HPTs were used to diagnose pregnancy and can thus be expected to have the highest use after the first 2 weeks following the first missed period, that is, 6–8 weeks of pregnancy (4–6 weeks embryonic development). This period corresponds to the time when the embryo is highly susceptible to developing central nervous and limb reduction defects. Exposure to HPTs after the second missed period (10 weeks gestation or 8 weeks embryonic development) and exposure even later may also have happened, for example, in women with irregular menstruation. This implies that pregnant women could have been exposed to HPTs during the entire organogenic period, that is, weeks 4–12 of pregnancy (2–10 developmental weeks). All the types of malformations reported associated with HPTs in case reports and in epidemiological studies (see Section 3.1) can also be induced during this time. The fifth criterion is therefore considered to be met.

### 3.4 Criteria 6: Evaluation of a Plausible Mechanism

The concept of biologic plausibility is central to the discussion of evidence. Evidence can be drawn from mechanisms of action of a drug and recognizing a pattern of malformations to establish that a cause–effect relationship is biologically plausible.

The almost identical pattern of malformations associated with HPTs and the established human teratogen misoprostol in the case of failed abortion strongly indicates a common etiology for their teratogenicity. Furthermore, the abortifacient drug mifepristone has, despite limited use as an abortifacient alone, been associated with similar malformations. Together, these data suggest that the pathogenesis of the teratogenicity in humans for abortifacient drugs and HPTs are initiated via a common indirect effect, failed abortion, in some of the exposed pregnancies and not a direct effect of the substances in the embryo.

The primary effect of HPTs in laboratory animal studies is embryolethality when given during the period when HPTs were used in humans, and no congenital malformations were induced (comprehensive review by Clements 2016). Genital tract abnormalities and virilization of female fetuses can be induced in rodents and primates exposed to high doses of these hormones later during the period of sexual differentiation and are likely to be related to weak androgenic properties of norethisterone (Clements 2016).

To evaluate a possible common indirect mechanism initiated via failed abortion, it is relevant to present further information on 1) the ability of misoprostol, mifepristone, and HPTs to pharmacologically cause failed abortion and teratogenicity and 2) mechanistic data on how periods of hypoxia and ROS generation in the embryo can cause teratogenicity.

#### 3.4.1 Pharmacological Effects on the Uterus and Associated Risks for Human Teratogenicity

*Misoprostol* is a prostaglandin. Uterine contractions and abortions by misoprostol are mediated by a direct effect on prostaglandin receptors in the myometrium of the human uterus. As mentioned previously, several extensive studies indicate malformations associated with misoprostol to be causally related to uterine contractions, compression of utero‐placental and/or embryonic arteries, and subsequent hypoxia‐related teratogenicity in the embryo if abortion fails (Auffret et al. 2016; Gonzalez et al. 1998; Orioli and Castilla 2000). The success rate of abortion after misoprostol use alone varies between 82% and 100% (average around 90%) and depends on the route of administration and dosing schedule (Moseson et al. 2024). Only a minority of the pregnancies (~10%) will thus show failed abortion. It is only among these pregnancies that there is a risk of teratogenicity.

It has been estimated that the risk for teratogenicity after use of misoprostol to terminate pregnancy is low and does not exceed 10 malformations per 1000 pregnant women (Philip et al. 2003). These data, taken together, indicate that around 10% of the women with failed abortion experience uterine contractions and embryonic hypoxia of such magnitude that teratogenicity is induced.

*Mifepristone* was developed in the 1980s as a human abortifacient drug to be used alone. The mechanism for the abortive effect is related to the antagonistic effect of mifepristone on progesterone receptors. Mifepristone has a 2‐fold higher affinity than progesterone for these receptors, resulting in a decreased receptor‐mediated relaxation effect on the uterus and production of uterine contractions and abortion (Bygdeman and Swahn 1985). However, as with misoprostol, successful abortion does not occur in all women (Spitz and Robbins 1998). In fact, the response varied from 64% to 73% and was most effective in women with lower pretreatment levels of progesterone (Birgerson and Odlind 1988) and at early gestational ages in the first trimester (Spitz and Robbins 1998), again when progesterone levels are lower. In one study, when the mean gestational age was week 14, the abortion rate was only 10% (Bernard et al. 2013). Because of these results, mifepristone ceased to be used as a single abortifacient drug and is today used in combination with a prostaglandin, such as gemeprost or misoprostol. The most common prescription today is mifepristone given a few days before misoprostol. This results in a successful abortion rate of 95% or higher.

There are, therefore, only a few pregnancies exposed to mifepristone alone and instances where women took mifepristone but then decided not to proceed with the termination. Despite the low number of pregnancies exposed, and even fewer cases where pregnancy continued after failed abortion, limb and nervous defects typical for misoprostol have been reported after single use of mifepristone. A review of adverse events reported to FDA, with 13 documented cases of failed abortion, showed that 3 out of 13 were diagnosed with serious malformations (Gary and Harrison 2006). The malformations consisted of one case of Moebius syndrome, one case with a neural tube defect, and a third case with a neural tube defect combined with finger amputation defects and facial dysmorphia. Other reported malformations in case reports in the literature include sirenomelia combined with anal atresia, renal agenesis, and cleft lip and palate (Pons et al. 1991) and a case with limb amputation, club foot, and cerebellum atrophy (Sentilhes et al. 2007). Another study in France (Bernard et al. 2013) reported one case of hydrocephaly combined with a thumb defect and a second case with disruption of the sympathetic nerve supply in the eye region after failed abortion.

Human data is too limited to determine whether mifepristone is a human teratogen by using traditional statistical methods. However, the occurrence of rare malformations, for example, Moebius syndrome (Bavinck and Weaver 1986), limb reductions, and neural tube defects of a similar type reported after use of misoprostol indicates a common teratogenic mechanism. The only published teratology study in a human‐relevant animal model with a design that mimics how abortifacient drugs were administered in humans strongly supports this assumption (Jost 1986). Mifepristone produced cranial and neural tube malformations, eye defects, and limb reduction defects in pregnant rabbits showing failed abortion. Together, the results indicate that mifepristone, via its anti‐progesterone effect, can cause malformations secondary to failed abortion. This is also reflected in regulatory pregnancy labeling. Like misoprostol (Misoprostol Healthcare Professional (SmPc) 2021), pregnancy texts warn that if the patient chooses to continue the pregnancy despite a failed abortion using mifepristone, then the pregnancy should be followed carefully with a focus on malformations, particularly limb defects (Mifepristone Healthcare Professional (SmPc) 2024).

*Hormone pregnancy tests*. The almost identical pattern of malformations associated with the use of HPTs and misoprostol (see Table 3) strongly indicates that HPTs, like misoprostol, have the potential to cause human teratogenicity via induction of uterine contractions and a failed abortion process in some exposed pregnancies. As mentioned in the Introduction, the principle of action of HPTs was to artificially produce a sudden high peak of the synthetic progesterone norethisterone (NET) and high progesterone receptor stimulation, followed by a decrease in progesterone receptor stimulation. This mimics normal menstruation and results in uterine contractions and expulsion of the endometrium in non‐pregnant women.

When the HPT principle was introduced in the late 1950s, it was claimed that an existing pregnancy was not affected since pregnant women had higher levels of progesterone than non‐pregnant women. However, today, it is known that wide variations exist in progesterone levels in both non‐pregnant and pregnant women. During the menstrual cycle, progesterone levels rise from 0.1–0.7 to 2–25 ng/mL at ovulation followed by a decrease if the egg is not fertilized. In pregnancy, progesterone produced by the corpus luteum and later by the placenta continues to increase, but levels may vary considerably in early pregnancy (range 11–44.3 ng/mL). This means that progesterone levels in some pregnant women can be equal to or lower than those in some non‐pregnant women (Ku et al. 2021; Rochester Medical Center 2024).

The results imply that an abortion process might also be initiated by HPTs in some pregnant women with progesterone levels lower than those of non‐pregnant women. This assumption is supported by a study following corpus luteum removal resulting in the loss of the progesterone receptor–mediated relaxant effect on the uterus with concomitant increased uterine activity and abortion (Csapo et al. 1974). The degree of progesterone withdrawal and abortive effect was a function of gestational age. Women who were lupectomized 12 days after a missed period aborted within 2–3 days; after 21 days, within 4–5 days; and after 30 days, no abortion occurred (Csapo et al. 1974). This suggests that the abortive effect produced by decreased progesterone receptor stimulation (progesterone withdrawal) diminishes with increasing progesterone levels as progesterone levels rise during pregnancy.

The main metabolite of norethisterone, 5 alpha‐NET, may be a contributing factor to the initiation of an abortion process after the use of HPTs containing NET. This metabolite has the same anti‐progesterone effects as mifepristone and has a 2‐fold higher binding capacity to progesterone receptors compared to progesterone (Castro et al. 1995). This means that progesterone receptors prefer to bind to the progesterone antagonist 5 alpha‐NET instead of progesterone. This also means that, like mifepristone, pregnant women with low levels of progesterone are likely to be more susceptible to the abortive effect of 5 alpha‐NET. This metabolite may also reach high concentrations in some situations, for example, using higher doses than therapeutic and in women with low metabolizing capacity of the enzyme 5‐alpha‐reductase since further metabolism via this enzyme is a rate‐limiting step (Blom et al. 2001).

No formal clinical trials were required by the manufacturer before HPTs were used in pregnant women. However, a human clinical trial with HPTs supports the hypothesis that failed abortion occurred in some pregnant women using HPTs (Rawlings 1960). Five out of 66 women with 5–8 weeks amenorrhea using HPTs for 2 days showed neither menstruation bleed (“not pregnant”) nor absence of bleeding (“pregnant”); rather, they showed “spotting” (manifested as brown staining) and signs similar to an early threatened abortion. Three of these women were subsequently proven to be pregnant. Thus, three of 35 pregnant women (8.6%) using HPTs showed clinical signs of early threatened/failed abortion with the pregnancy continuing. There are plausible pharmacological mechanisms by which HPTs would induce failed abortion in a small percentage of the pregnant women, as discussed previously; pregnant women with lower progesterone levels than average may be at risk.

These data also indicate that the great majority of pregnancies were unaffected after the use of HPTs at recommended doses; only a small percentage (8.6%) showed failed abortion. Based on estimations for misoprostol, a small percentage of pregnancies with failed abortion (10%) would result in malformations. Uterine contractions in women with failed abortion after misoprostol use (at doses causing complete abortion in most pregnancies) may on average have been more severe and resulted in a higher risk for teratogenicity than in women with signs of failed abortion after HPT use. If the teratogenic risk is estimated to be 2‐ to 3‐fold lower with HPTs compared to misoprostol in the case of failed abortion by a conservative approach, this suggests a 3%–5% risk for malformations in women with failed abortion after the use of HPTs.

There is, however, no “safe dose” after using misoprostol use, and even low doses used to prevent peptic ulcer after the use of NSAIDS can cause typical misoprostol teratogenicity (Auffret et al. 2016; Vauzelle et al. 2013). Furthermore, the very similar teratogenicity shown for HPTs and misoprostol strongly indicates a failed abortion mechanism (but the embryo survives) underlying the teratogenicity of HPTs. Due to the widespread use of HPTs, these data indicate that a substantial number of women gave birth to malformed children as a direct consequence of their HPT use, despite the risk in an individual pregnancy being low. This aspect is discussed in Section 3.5.

#### 3.4.2 Mechanisms by Which Periods of Interrupted Oxygen Supply Can Cause Teratogenicity

Human and animal data indicate that embryonic hypoxia and the generation of ROS secondary to a failed abortion process could provide an explanation for the individual malformations associated with HPTs and abortive drugs.

ROS generated in the endothelium of recently formed vessels in the embryo during hypoxia, particularly when oxygen returns (re‐oxygenation) to hypoxic tissues, are able to cause malformations secondary to local damage of the vascular system in the embryo, so‐called “vascular disruption” defects (Auffret et al. 2016; Danielsson et al. 2023; Davies 1995; Philip et al. 2003; Vargas et al. 2000). Hypoxia‐related ROS generation is also able to cause severe cardiac rhythm disturbances (including periods of cardiac arrest) in the embryo during the long period of cardiogenesis. This can result in cardiovascular defects, as will be discussed below.

*ROS‐induced malformations due to vascular disruption*. The formation of the vascular system is essential for the normal development of all other organ systems; all anatomical structures are dependent on an intact vascular supply for adequate oxygen and nutrient supplies. In contrast to adult vessels, the vasculature in developing embryonic tissues is characterized by a high proliferation rate, resulting in fragile, thin vessels with minimal smooth muscle reinforcement, making this type of vasculature a target for ROS‐induced disruption (Danielsson et al. 2023). There is a delay in the formation of the endothelium layer and the formation of the protective muscle layer of vessels in the early embryo (Vargesson 2003). Thus, newly formed vessels are particularly vulnerable; ROS exposure results in small vessel destruction and hemorrhage while angiogenesis remains intact in more mature vessels (Danielsson et al. 2023). Local vascular damage with hemorrhage (vascular disruption) results in the maldevelopment of anatomical structures that should have been supplied by the damaged vessel. Consequently, vascular disruptions can cause specific defects and patterns of birth defects depending on the location, extent, and timing of the vascular disruptive event in embryonic life.

Many of the malformations associated with misoprostol and HPTs, including limb reduction and neural tube defects, and cleft lip/palate, can be induced by a single period of embryonic hypoxia in rats, mice, and/or rabbits (Danielsson et al. 2023; Ritchie et al. 2017; Webster and Abela 2007). These defects are preceded by edema, vascular disruption, and hemorrhage in embryonic tissues shortly after the hypoxic event (Danielsson et al. 2023) and are likely to be ROS‐induced (Chernoff and Rogers 2010). Also, other defects such as unilateral absence of kidney, abdominal wall defects, skeletal defects—including vertebral, rib, and mandibular defects; syndactyly; anal atresia; and defects included in VACTERL (e.g., vertebral, anal atresia, renal, and limb defects)—can be induced by a single period of embryonic hypoxia (Danielsson et al. 2023; Sköld et al. 2001; Wellfelt et al. 1999).

There are also several human studies discussing hypoxia and/or vascular disruption as a mechanism to explain the pathogenesis of malformations associated with the use of HPTs, such as limb reductions, cleft lip/palate, unilateral kidney agenesis, nervous system defects, including neural tube defects (Stevenson et al. 1987; Van Allen 1981, 1992), syndactyly (Philip et al. 2003), esophageal atresia (Ingalls and Prindle 1949), anorectal atresia (Stevenson and Hunter 2013), and VACTERL association (Stevenson et al. 1987). There is also considerable evidence that some rare multiple defects (Moebius, Poland, and Klippel–Feil association) are related to embryonic hypoxia due to temporary compression of different branches of the subclavian artery in the embryo (Bavinck and Weaver 1986). Vascular disruption–related rib agenesis of two ribs on the left side is associated with dextrocardia. A proposed mechanism is mechanical intrauterine displacement of the heart due to insufficient protection of the chest wall against external pressures (Torre et al. 2010). HPTs showed a 2‐fold increase in dextrocardia (Table 2). Thus, vascular disruption could explain many of the individual malformations associated with HPTs.

*ROS‐induced cardiac arrhythmia and cardiovascular defects*. The immature embryonic heart, from when the tubular heart starts beating until the four‐chamber heart has been formed, seems to be highly susceptible to hypoxia/ROS generation. Studies in human‐relevant vertebrate experimental models show that when exposed to transient hypoxia, the immature embryonic heart responds with severe rhythm disturbances, for example, bradycardia and cardiac arrest (Chernoff and Grabowski 1971; Grabowski and Schroeder 1968), in a similar way as shown in adults (Granger and Kvietys 2015; Hearse and Tosaki 1987; Tanaka and Hearse 1988). The induced cardiovascular defects associated with HPTs and misoprostol during the long period of cardiogenesis are likely to be related to the induced rhythm disturbances. A wide variation of cardiovascular defects similar to those associated with HPTs in humans can be induced by selectively increasing, decreasing, or misdirecting blood flow in the developing cardiovascular system by mechanical methods or by cardioactive substances (Midgett et al. 2017; Sedmera 2005; Trinidad et al. 2022).

Mechanistic studies indicate that the induced arrhythmias are caused by hypoxia/ROS‐mediated inactivation of ion channels, particularly the potassium channel (IKr) (Nanduri et al. 2009; Taglialatela et al. 1997; Wang et al. 2004, 2016). This channel is of major importance for cardiac rhythm regulation across species, including the human embryo (Danielsson et al. 2013). It is possible to block this channel pharmacologically, thereby mimicking the consequences of hypoxia/ROS‐induced cardiac arrhythmia in the embryo. By blocking this channel during different stages of organogenesis in rat embryos, a spectrum of cardiovascular anomalies can be induced. The spectrum includes transposition of vessels (e.g., retroesophageal right subclavian artery), absence of vessels (e.g., lack of innominate), abnormal vessels (e.g., enlarged aorta with reduced pulmonary trunk, overriding aorta) and cardiac interventricular septum defects. These defects can be induced by a selective IKr blocker in rats during a period corresponding to weeks 4–8 of human pregnancy (Sköld et al. 2001; Wellfelt et al. 1999).

In addition to inducing hypoxia‐induced cardiovascular defects, potent IKr‐blocking drugs can cause typical stage‐specific hypoxia‐vascular disruption‐related malformations (Danielsson et al. 2003). There is also evidence that situs inversus can be induced by hypoxia/ROS mechanisms (Danielsson et al. 2023). ROS‐induced severe irregular cardiac rhythm in the embryo could aggravate embryonic hypoxia produced by compression of uterine vessels, for example, HPTs. This could explain how even a very short period of hypoxia (minutes) can produce severe malformations in embryonic tissues by inducing cardiac arrest/embryonic cardiac arrhythmia (Danielsson et al. 2023). Pretreatment with a substance that captures ROS before administration of the IKr blocker almost completely prevented the occurrence of both vascular disruption defects as well as cardiovascular defects (Sköld et al. 2001; Wellfelt et al. 1999). These results present strong evidence for a hypoxia–ROS‐mediated effect underlying “failed abortion teratogenicity” both for cardiovascular and vascular disruption anomalies.

Altogether, this evaluation presents evidence that HPTs have the potential to induce teratogenicity via failed abortion and periods of hypoxia/ROS. The results show that criterion 6 “plausible mechanism” is fully met.

### 3.5 Criterion 7: Increases of Anomalies in a Population After Introduction of HPTs

HPT exposure in the first trimester appears to have been common during a restricted period in the 1970s in some countries, for example, in the UK (Ord and Wooley 2016). During the period 1970–1977 (8 years), around 5.37 million live births in total were registered in England and Wales (Commission on Human Medicines 2017). During this time, it is estimated that HPTs were used as pregnancy tests in 971,482 pregnancies. As discussed in Section 3.4, only pregnancies showing failed abortion (estimated 8.6% for HPTs) are at risk for teratogenicity, and among those with failed abortion, only a small percentage (3%–5%) are estimated to develop malformations. Thus, 83,547 pregnancies can be estimated to have shown signs of failed abortion and 2506‐4177 to show major malformations. This figure can be compared to the background risk for major malformations in EUROCAT registers of 2.3% (Dolk 2015). The number who would be expected to have given birth to a child with a congenital anomaly in the UK from 1970 to 1977 would be about 123,000 in the general population. The anomalies associated with HPTs exposure, including common anomalies such as orofacial clefts, are thus likely to account for a relatively small proportion of all birth defects during this period (2%–3%). In addition, the lack of documentation of increases and decreases in the number of reported anomalies after the introduction and restriction of HPTs in the UK (and other countries in the 1960s and 1970s) does not lend additional support in the evaluation of HPTs teratogenicity.

### 3.6 Criteria 8: Animal Studies

#### 3.6.1 Principles to Evaluate Potential Human Risk Based on Results in Animal Studies

To fully characterize the potential human developmental toxicity of an agent, conducted animal studies should be evaluated according to principles expressed in a global guideline on reproductive toxicology testing (ICH S5 (R3) 2020). These principles stress addressing any limitations and their impact on the used animal models (most often rats, mice, rabbits and/or monkeys). Other important aspects to evaluate are design, doses, exposures, and dosing schedules in conducted animal studies compared to human dosing. Also, the consistency of findings reported between species can strengthen the concern for a similar adverse effect in humans. In order to properly assess potential adverse effects, including malformations, a sufficient number of litters, at least 16 litters in rodents and rabbits, and at least 16 fetuses in monkeys is expected (ICH S5 (R3) 2020).

The ICH S5 R3 guideline also mention, “further knowledge of the mechanism of reproductive or developmental effects can help to explain differences in responses between species” and that “a finding that can be interpreted as a consequence of pharmacology suggests that it will be of concern for humans.” Human studies indicate a common pharmacologically induced failed abortion mechanism underlies the similar malformations reported for HPTs, misoprostol, and mifepristone. To further investigate a common mechanism, it is therefore important to compare the results in teratology studies for these substances with special attention to known species similarities/differences in abortive potential between humans and the species used in teratology studies.

#### 3.6.2 Rats: Effects of HPTs and Abortifacient Drugs in Teratology Studies

The process of pregnancy termination essentially differs between humans and rodents. Rodents do not expel embryos by uterine contractions (i.e., abort the embryo) during the period of organogenesis (GD 6‐17 in rats and GD 6‐15 in mice). Instead, embryos are gradually degraded and then resorbed into the uterus (Clements 2016; Danielsson et al. 2023; Macfarlane et al. 1957). This means that the human failed abortion mechanism for induction of malformations in organogenesis does not exist in rats and mice during organogenesis. This likely explains the absence of teratogenicity after dosing in organogenesis (whole or part) in rodents despite the administration of high doses with HPTs (Clements 2016), mifepristone (Mifepristone Healthcare Professional (SmPc) 2024) or misoprostol (Kotsonis et al. 1985). Instead, pronounced dose‐related inccreases in resorptions starting at doses similar to human therapeutic doses were recorded for both HPTs (Clements 2016) and mifepristone (Mifepristone Healthcare Professional (SmPc) 2024). Both these agents act on progesterone receptors. The high increases in HPT‐induced embryonic death are most likely related to the withdrawal/antagonism of progesterone's pregnancy‐maintaining effect from implantation and onwards on progesterone receptors (Clements 2016).

#### 3.6.3 Rabbits: Effects of HPTs and Abortifacient Drugs in Teratology Studies

In conventional teratology studies, rabbits are dosed during organogenesis (GD 6‐19). Similar to rats, rabbits terminate pregnancy by resorption in early organogenesis up to around GD 10 (Chang et al. 1993). However, from around GD 11‐12, rabbits are able to both abort and resorb embryos for a few days, while only abortions occur after GD 14 (Ozalp et al. 2008). In conventionally designed teratology studies, a dose‐related increase in resorptions, with almost all embryos dead at higher doses, was recorded for both HPTs (Clements 2016) and mifepristone (Mifepristone Healthcare Professional (SmPc) 2024). The pregnancy‐terminating effect for HPTs started at doses equivalent to human therapeutic doses. Follow‐up studies with HPTs (with similar doses as in conventional teratology studies) showed that high incidences of embryonic death were induced by dosing at an early stage of organogenesis, on GD 6‐7, 8‐9, or 9‐10 (Clements 2016). These data indicate that in conventional teratology studies in rabbits, embryonic death occurred during the stages when the rabbits can abort (before GD 11‐12) but teratogenicity was only induced after GD 12 by a failed abortion mechanism.

In contrast, when replicating the human dosing regimen with the administration of oral doses in rabbits at a stage when rabbits can abort (GD‐12‐15), both mifepristone and HPTs were associated with teratogenicity. This period corresponds to around weeks 5–8 of human pregnancy. Malformations were induced at a subtherapeutic dose for mifepristone (Jost 1986) and at a human equivalent dose for HPTs (Clements 2016). These doses allowed the survival of enough embryos to evaluate teratogenicity; higher doses resulted in pronounced embryonic death. Dosing in a study with 40 pregnant rabbits starting on GD 11 with mifepristone for a few days (1, 2, 3, 4 or 5 days) induced neural tube, limb reduction, and eye defects in animals showing failed abortion (Jost 1986).

In a similar manner, dosing with HPTs for 2 days on GD 12‐13 or GD 14‐15 resulted in fetuses with neural tube defects and others with major external/visceral malformations, while dosing for 2 days before GD 12 (GD 6‐7, 8‐9, 10‐11) did not induce any malformations (Clements 2016). The neural tube defects are significant since no neural tube defects were observed in pregnant control rabbits in the study (in total > 70 control litters evaluated). Neither were any neural tube defects observed in two large conventionally designed teratology studies in rabbits with three HPT dose groups plus a control group (16–22 L per group). In total, more than 100 litters were evaluated, resulting in dose‐related massive embryonic death (Clements 2016).

Finally, misoprostol has been shown to cause neural tube defects, as well as vertebral and abdominal wall defects in rabbits in a conventionally designed study (Clemens et al. 1997). It is highly likely that these malformations were also caused by failed abortion after GD 12; misoprostol is not expected to affect embryonic development before the uterus in rabbits is able to contract and induce abortion on and after GD 12.

#### 3.6.4 Monkeys: Effects of HPTs and Abortifacient Drugs in Teratology Studies

Non‐human primates are the best animal models to study effects of sex steroids on human reproduction, because of the similarity of reproduction (Henck et al. 1996). Unlike rats and rabbits, monkeys, like humans, terminate pregnancy via an abortion process mediated via uterine myometrium contractions during the whole of organogenesis. The pregnancy text for mifepristone mentions that abortions occurred at a high rate in conventionally designed studies (repeated oral dosing during whole organogenesis GD 20‐50) in monkeys (Mifepristone Healthcare Professional (SmPc) 2024). The abortions started at doses equivalent to the human therapeutic doses and the extent of abortion was so high that “the number of foetuses surviving the abortifacient action of mifepristone was insufficient for a conclusive assessment” (Mifepristone Healthcare Professional (SmPc) 2024). The prostaglandin misoprostol has not been studied in teratology studies in monkeys, however mifepristone in combination with misoprostol is effective in terminating pregnancy via abortion in monkeys (Micks et al. 2012).

The results in three teratology studies with a conventional design with the constituents in the most used HPT in the UK (Primodos) in Rhesus, baboons, and Cynomolgus monkeys show a dose‐dependent increase in abortion rate (Clements 2016). In Rhesus monkeys, abortions started to occur at doses equivalent to human HPT doses. A high incidence (up to 67%) of abortions was observed in all three species at doses greater than human therapeutic doses (Clements 2016). The abortion in baboons dosed with norethisterone was associated with a marked decrease in progesterone levels (Beck and Pope 1982). In rhesus monkeys, the abortion rate was higher when dosing was limited to early organogenesis compared to exposure only during late organogenesis (GD 33‐46), suggesting that the abortion rate is highest when progesterone levels are relatively low in early gestation (Prahalada and Hendrickx 1983).

Due to the high incidence of abortions in conventionally designed teratology in monkeys, the numbers of fetuses per HPT dose group examined at the cesarean section were low (4–10 per group). These numbers are much lower than the minimum number (16 fetuses) ICH S5 R3 requires to detect possible teratogenic potential. Nevertheless, one of the four examined fetuses in the low‐dose Cynomolgus group showed vertebral malformations of a similar type (rib and vertebral malformations) as associated with failed abortion after the use of HPTs in humans.

#### 3.6.5 Integration of Results in Pregnant Animals

The results show that HPTs can terminate pregnancy in a consistent manner across species (mice, rats, rabbits, and monkeys). The pregnancy termination potential of HPTs, manifested as resorptions in rodents and early gestational rabbits, and by abortions in monkeys and in rabbits (at later stages of organogenesis), was dose‐dependent and started at human equivalent doses. All these species are dependent on progesterone for maintaining pregnancy. Similar to the human abortifacient drug mifepristone, the pregnancy termination effect of HPTs is highly likely to be caused by the withdrawal/inhibition of the action of progesterone on progesterone receptors.

The studies also show that only animal species that can abort the embryo in organogenesis can be used to evaluate the potential risk for human teratogenicity caused by failed abortion. This explains why misoprostol, mifepristone, and HPTs are not teratogenic in rodents. Furthermore, the inhibition of the pregnancy‐maintaining effect of progesterone by HPTs and mifepristone in rabbits and monkeys in conventional teratology studies (dosing during whole organogenesis from implantation) resulted in high incidences of embryonic death shortly after the start of dosing. Consequently, the number of fetuses surviving the abortifacient action of HPTs was insufficient for a conclusive assessment of teratogenicity. Altogether, this also shows that conventionally designed animal teratology cannot be used to evaluate the risk for human teratogenicity by drugs with the potential to initiate an abortion process in humans.

In contrast, a dosing regimen in pregnant rabbits, which mimics the human with dosing for a few days with human equivalent doses during GD 12‐15 (when rabbits can abort), induced similar teratogenicity as observed in humans for both HPTs and mifepristone. This period in rabbits correspond to around human pregnancy week 5–8. The teratogenicity observed in rabbits show that the teratogenicity associated with HPTs can be recreated in an animal model which abort in organogenesis. Together, by addressing limitations and their impact on the used animal models and differences in doses/dosing schedules in humans versus animal studies, the results, provide evidence for human teratogenic potential of HPTs by a pharmacologically induced failed abortion mechanism. The criterion “recreation of human teratogenicity in animal studies” (criterion 8) is thus met.

## 4 Conclusions

To our knowledge, no study using pre‐defined criteria for the evaluation of the teratogenicity of HPTs has been published before. The criteria used for HPTs were the same as for misoprostol when misoprostol was evaluated to be a human teratogen. When applying these criteria for HPTs, the evaluation showed that HPTs meet the requirements for being causally associated with HPTs and thus should be considered teratogenic in humans.

The anomalies, which fulfill clinical criteria for a causal association between HPTs and human teratogenicity, consist of a spectrum of malformations:

- Limb reductions
- Neural tube defects
- Atresia/fistula of the esophagus
- Cleft lip with or without cleft palate
- Transposition of great vessels
- Urinary bladder exstrophy
- Renal agenesis
- VACTERL association.

The strikingly similar pattern of malformations (including the above‐mentioned anomalies) in human pregnancies exposed to HPT and misoprostol, together with other presented mechanistic and animal data, suggests a common failed abortion mechanism for their teratogenicity. Abnormal uterine contractions in connection with the initiation of an abortion process, resulting in compression of uterine/embryonic vessels and periods of hypoxia and ROS generation in the embryo (but the embryo survives), are likely to underlie their teratogenicity.

The type of malformation(s) induced is related to the stage of pregnancy when the hypoxic event occurred and the severity and duration of the hypoxic event. Mechanistic studies in pregnant animals exposed to a period of hypoxia support the hypoxia–ROS‐related mechanism. Almost all of the subgroups of anomalies associated with HPTs and misoprostol in humans have been recreated in animal studies by short periods of interruption of oxygen supply to the embryo; for example, clamping of uterine vessels or decreasing the oxygen content in the atmosphere (Danielsson et al. 2013). Pretreatment with a ROS capturing agent before the hypoxic event prevented teratogenicity induction, suggesting teratogenicity on the molecular level to be related to ROS generation.

The risk in this study was estimated to be small for an individual pregnancy exposed to HPTs (around 3–5 HPT‐induced malformations out of 1000 exposed to HPTs); women with low progesterone levels seem to have a higher risk. However, the extensive use of HPTs, with more than 20% of all pregnancies exposed to HPTs in the early 1970s, suggests that around 2500 to 4200 women gave birth to a malformed child caused by HPTs during the period 1970–1977 in the UK only.

## Data Availability

Data sharing is not applicable to this article as no new data were created or analyzed in this study.
